# Electronic feedback in a diet- and physical activity-based lifestyle intervention for weight loss: a randomized controlled trial

**DOI:** 10.1186/1479-5868-8-41

**Published:** 2011-05-18

**Authors:** Sara L Shuger, Vaughn W Barry, Xuemei Sui, Amanda McClain, Gregory A Hand, Sara Wilcox, Rebecca A Meriwether, James W Hardin, Steven N Blair

**Affiliations:** 1 Salem College, 601 S. Church St., Winston-Salem, NC 27101, USA; 2 Department of Exercise Science, University of South Carolina, 921 Assembly St., Columbia, SC 29208, USA; 3 Division of Nutritional Sciences, Cornell University, Ithaca, NY 14850, USA; 4 Department of Epidemiology and Biostatistics, University of South Carolina, 800 Sumter St., Columbia, SC 29208, USA

## Abstract

**Background:**

The SenseWear™ Armband (SWA) (BodyMedia, Inc. Pittsburgh, PA) is a physical activity and lifestyle monitor that objectively and accurately measures free-living energy balance and sleep and includes software for self-monitoring of daily energy expenditure and energy intake. The real-time feedback of the SWA can improve individual self-monitoring and, therefore, enhance weight loss outcomes.

**Methods:**

We recruited 197 sedentary overweight or obese adults (age, 46.8 ± 10.8 y; body mass index (BMI), 33.3 ± 5.2 kg/m^2^; 81% women, 32% African-American) from the greater Columbia, South Carolina area. Participants were randomized into 1 of 4 groups, a self-directed weight loss program via an evidence-based weight loss manual (Standard Care, n = 50), a group-based behavioral weight loss program (GWL, n = 49), the armband alone (SWA-alone, n = 49), or the GWL plus the armband (GWL+SWA, n = 49), during the 9-month intervention. The primary outcome was change in body weight and waist circumference. A mixed-model repeated-measures analysis compared change in the intervention groups to the standard care group on weight and waist circumference status after adjusting for age, sex, race, education, energy expenditure, and recruitment wave.

**Results:**

Body weight was available for 62% of participants at 9 months (52% standard care, 70% intervention). There was significant weight loss in all 3 intervention groups (GWL, 1.86 kg, P = 0.05; SWA-alone, 3.55 kg, P = 0.0002; GWL+SWA, 6.59 kg, P < 0.0001) but not in the Standard Care group (0.89 kg, P = 0.39) at month 9. Only the GWL+SWA group achieved significant weight loss at month 9 compared to the Standard Care group (P = 0.04). Significant waist circumference reductions were achieved in all 4 groups at month 9 (Standard Care, 3.49 cm, P = 0.0004; GWL, 2.42 cm, P = 0.008; SWA-alone, 3.59 cm, P < 0.0001; GWL+SWA, 6.77 cm, P < 0.0001), but no intervention group had significantly reduced waist circumference compared to the Standard Care group.

**Conclusions:**

Continuous self-monitoring from wearable technology with real-time feedback may be particularly useful to enhance lifestyle changes that promote weight loss in sedentary overweight or obese adults. This strategy, combined with a group-based behavioral intervention, may yield optimal weight loss.

**Trial Registration:**

ClinicalTrials.gov: NCT00957008

## Background

The prevalence of obesity in the US continues to be high, exceeding 30% in most sex and age groups [1]. Obesity is a risk factor for various chronic conditions including diabetes, hypertension, high cholesterol, stroke, heart disease, and certain cancers [2]. Preventing and treating obesity is a multifaceted dilemma that will likely need to be addressed on multiple levels ranging from policies to individual interventions.

Clinical and commercial weight loss interventions can produce short-term weight loss, but a majority of people regain about 40% of their lost weight in the first year and continue to regain over time [3]. Examining effective approaches to weight loss may be helpful for determining the behavioral skills needed to produce weight loss and weight maintenance. Self-monitoring has emerged as a key skill for weight management, because those who weigh daily or weekly have greater success attaining weight loss goals [4]. Self-monitoring increases awareness of energy intake and expenditure, enhances self-efficacy, and allows for individuals to monitor progress and change over time [5]. It might also facilitate regular goal setting. However, barriers such as stress, lack of social support, and lack of time can potentially affect adherence to self-monitoring [5].

The SenseWear Armband (SWA) (BodyMedia, Inc. Pittsburgh, PA) is a physical activity monitor that is worn on the upper arm and works with an online website for self-monitoring of daily energy expenditure and energy intake. The SWA also works with a watch-like display device that gives the user real-time feedback on calories burned, steps taken, and minutes of physical activity. These data are collected by the SWA and can improve individual self-monitoring and, therefore, enhance weight loss outcomes. The SWA device is a commercially-available self-monitor (http://www.bodymedia.com) that is priced comparably to other activity monitors. The primary form of treatment for obesity is behavioral therapy, an approach that combines education about diet and exercise with behavioral strategies that support behavior change [6,7]. The SWA could increase individuals' adherence to healthy lifestyle goals by promoting more continuous self-monitoring to improve weight loss, due to the automated nature and real-time feedback of the device.

The aim of this study was to determine the effectiveness of continuous self-monitoring and feedback from technology (achieved through the automation of the SWA) alone and in combination with GWL (group weight loss) to enhance weight loss and waist circumference reduction over a 9-month period in sedentary overweight or obese adults. We hypothesized that an intervention that incorporated group weight loss sessions with continuous self-monitoring, a SenseWear Armband, interactive weight loss software, and a weight loss manual would produce greater weight loss and waist circumference reduction than a similar intervention that did not include the SenseWear Armband and continuous self-monitoring.

## Methods

### Study design

A complete description of the Lifestyle Education for Activity and Nutrition (LEAN) study design and methods is presented elsewhere [8]. In brief, the study was a randomized controlled trial with a Standard Care control group and 3 intervention groups. The research protocol was reviewed and approved annually by the University of South Carolina's institutional review board, and written informed consent was obtained from all participants prior to joining the study. Pre-post outcome data were collected from participants at baseline, month 4 and month 9. Participants were recruited in 3 waves.

### Study participants

**Figure 1:**
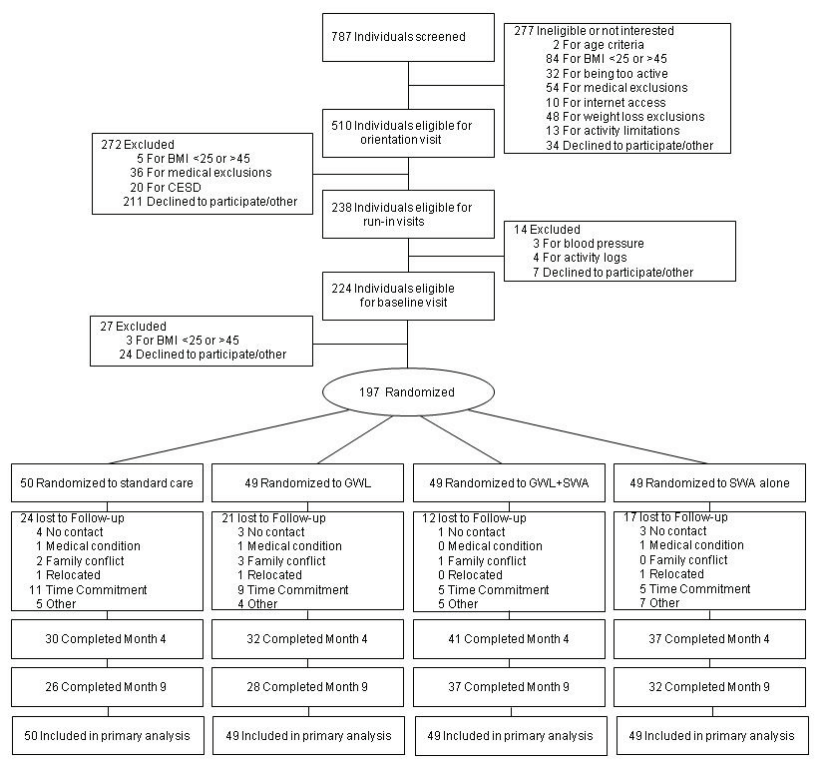
**Participant Flow Diagram**.

We conducted a total of 787 telephone screening interviews from the greater Columbia, South Carolina area between February 2008 and February 2009 (Figure 1). 197 men and women aged 18 to 64 years who were underactive (not accumulating 150 minutes of moderate-to-vigorous physical activity throughout the week in bouts ≥ 10 minutes), overweight or obese (body mass index ( BMI) = 25 - 45 kg/m^2^), and had access to the internet were randomly assigned to 1 of the 4 groups. Exclusion criteria included significant weight loss (>20 lbs) in the last 6 months, elevated blood pressure (160/95 mm Hg), ailments that limited physical activity, or serious medical conditions or other issues (e.g., pregnancy or depression) that contraindicated or confounded the weight loss intervention. Participants were recruited using a wide variety of techniques, including newspaper, mailers, community events, and worksite and other e-mail distributions.

### Standard Care (control group)

Standard Care participants received a self-directed weight loss manual based on two evidence-based programs, Active Living Every Day (ALED) and Healthy Eating Every Day (HEED) [9,10]. The manual's focus was to help individuals adopt a healthful eating pattern and increase their physical activity levels through the use of cognitive and behavioral strategies consistent with the Transtheoretical Model [11] and Social Cognitive Theory [12].

### Intervention groups

All study participants in the intervention groups also received the evidence-based weight loss manual that was given to the Standard Care group. The weight loss manual included two parts: a weight loss workbook included 14 chapters about healthy eating and active living and a set of forms for participants to use to record their daily meal and lifestyle activity, emotion or mood. Therefore, all the participants (including Standard Care) were instructed to self-monitor their dietary intake and physical activity.

#### Group-based behavioral weight loss education group (GWL)

During the first 4 months of the intervention, participants in this group received 14 GWL sessions from a facilitator based on ALED and HEED. Each session followed the ALED and HEED curriculum format, with the addition of a weekly weigh-in and greater emphasis on weight loss than in the original programs. During the final 5 months participants received 6 one-on-one telephone counseling sessions to provide continued support and enhance weight loss maintenance.

#### Armband alone group (SWA-alone)

The SWA-alone group received the SenseWear™ platform consisting of the armband, a real-time wrist watch display, and access to a personalized Weight Management Solutions web account. While wearing the armband, participants received real-time feedback from the wrist watch on several outcomes (i.e. energy expenditure, minutes spent in moderate and vigorous physical activity, and steps per day). Feedback regarding energy balance was received as participants regularly uploaded their armband to the website and recorded daily energy intake and body weight to the Weight Management Solutions web account. Participants were asked to wear the armband 16 hours a day, 7 days a week.

#### Combined GWL and SWA group (GWL+SWA)

These participants received all components of the GWL, including the 6 one-on-one telephone counseling sessions, as well as the SenseWear™ platform.

### Outcomes

The primary outcomes were body weight (kg) and waist circumference (cm). Secondary outcomes were BMI (kg/m^2^) and percent body fat. Body weight was assessed using a calibrated balance-beam scale. Heights were assessed using the same wall-mounted stadiometer. Waist circumference was measured using a standard protocol. BMI was calculated as weight in kilograms divided by height in meters squared. Body fat measures were assessed using standardized gender-specific 3-site skin fold measures. The male and female skin fold sites were the chest, abdomen, and thigh and the triceps, suprailiac, and thigh, respectively. Body density and percent body fat were estimated using the American College of Sports Medicine's gender-specific equations [13], followed by the Siri equation [14].

### Other measures

Physical activity assessments were taken at baseline and at month 9 (Table 1). During these assessments all participants wore the SenseWear™ Armband for 7 days without physical activity feedback. The SenseWear™ Armband is a lightweight physical activity monitor worn halfway between the acromion and olecranon processes on the upper left arm. This device uses four sensors to assess energy expenditure, sleep duration and efficiency, physical activity levels (sedentary, moderate, vigorous) and duration, steps, and on/off body wear time. The SWA includes a tri-axial accelerometer, a thermistor-based skin sensor, a proprietary heat flux sensor and a galvanic skin response sensor. This device has been shown to be valid in resting, exercise (i.e., treadmill and cycle ergometry), and free-living conditions (i.e., physical activity and exercise) [15-20]. Age, gender, race, and education were self-reported.

**Table 1:** Baseline Characteristics

Characteristics*	Total (n = 197)	Standard Care† (n = 50)	GWL† (n = 49)	SWA-alone† (n = 49)	GWL+SWA† (n = 49)
	(mean (SD) or N(%))	(mean (SD) or N(%))	(mean (SD) or N(%))	(mean (SD) or N(%))	(mean (SD) or N(%))
Age (years)	46.9 (10.8)	47.2 (8.9)	46.8 (12.4)	47.7 (11.6)	45.7 (10.4)
Female (%)	161 (81.7)	42 (84.0)	39 (79.6)	40 (81.6)	40 (81.6)
Race (%)					
White	131 (66.8)	30 (60.0)	33 (68.8)	33 (67.4)	35 (71.4)
Black	63 (32.1)	19 (38)	14 (29.2)	16 (32.7)	14 (28.6)
Other	2 (1.0)	1 (2.0)	1 (2.1)	0 (0.0)	0 (0.0)
College degree, 4 years (%)	152 (77.2)	37 (74.0)	40 (81.6)	39 (79.6)	36 (73.5)
Weight (kg)	92.8 (18.4)	94.2 (18.2)	93.2 (18.6)	92.0 (21.0)	91.9 (15.7)
Waist Circumference (cm)	99.7 (13.9)	100.1 (13.2)	101.2 (13.0)	98.5 (15.4)	98.9 (14.3)
Body mass index	33.3 (5.2)	33.7 (5.5)	33.1 (4.8)	33.2 (5.4)	33.0 (5.0)
% Body Fat	38.4 (5.3)	38.9 (4.7)	38.1 (5.8)	38.3 (5.3)	38.1 (5.3)
Mean Energy expenditure (kcals/day)‡	2209.4 (502)	2137.1 (416.0)	2277.3 (563.5)	2233.7 (542.9)	2185.5 (473.4)

*After bonferoni adjustments no significant differences were found.

† Standard Care: received a manual containing weight loss information; GWL: group-based behavior change weight loss program; SWA-alone: armband only; GWL+SWA: group-based behavior change weight loss program plus SWA.

‡Only available in 182 participants

### Participant retention and adherence

To reduce participant dropout and maintain adherence, several strategies were used, including a 2-week pre-randomization run-in period, behavioral contracts, and consistent support from staff members. Participants were reimbursed $10 for completion of baseline and month 4 follow-up assessments. Participants were reimbursed another $15 in incentives for completion of month 9 follow-up assessments.

### Randomization

Eligible participants were randomly assigned after completing run-in and baseline assessments. The randomization sequence was computer generated. The sequence was determined from randomly permuted blocks of equal length with fixed numbers of treatment allotments each, to balance treatment enrollments over time.

### Statistical Analysis

Given the target enrollment of 50 participants per treatment condition, the study design had 80% power to detect an effect size of 0.62 (assuming α = 0.025) for weight loss and waist circumference reduction. Under 40% attrition the study design had 80% power to detect an effect size of 0.81 (assuming α = 0.025) for weight loss and waist circumference reduction. If we assume a standard deviation of approximately 7.0 for the baseline follow-up differences for two outcome measures of interest, we had 80% power to detect a .434 kilogram difference in weight-loss and a .567 cm difference in waist size reduction.

Descriptive baseline characteristics of groups were tabulated as means and SDs or as percentages. Linear mixed models (SAS PROC MIXED) were conducted that controlled for age, race, gender, education, energy expenditure, and recruitment wave. A linear function for time was tested. All reported P values are 2-sided. All analyses were performed using SAS version 9.2 (SAS Institute Inc, Cary, NC).

## Results

Of the 197 randomized participants, 70% (58% of control and 72% of intervention) and 62% (52% control and 70% intervention) of the participants completed the month 4 and month 9 assessments, respectively (Figure 1).

As summarized in Table 1, the mean (SD) age of the study population was 46.9 (10.8) years; the mean (SD) BMI was 33.3 (5.2), and the mean (SD) waist circumference was 99.7 (13.9) cm. About 32% were nonwhite, and 82% were female. A majority of the participants (77%) had earned at least a college degree. As shown in Table 2, significant weight reductions were found in all 3 intervention groups at month 9 (all P ≤ 0.05). The SWA-alone (P = 0.003) and GWL+SWA (P < 0.0001) groups achieved significant weight loss at month 4. The Standard Care group did not show significant weight loss at either time point (i.e., month 4 or month 9). At month 4, there were no significant differences in weight among any of the three intervention groups compared with the Standard Care group (All p > 0.05). However, at month 9, the GWL+SWA group showed significant weight reduction when compared to the Standard Care group (P = 0.04).

**Table 2:** Least Square Means* across Time.

		Standard Care (n = 50)	GWL (n = 49)	SWA-alone (n = 49)	GWL+SWA (n = 49)
		(mean (s.e.))	(mean (s.e.))	(mean (s.e.))	(mean (s.e.))
Weight (kg)‡	BL	102.22 (2.97)	101.84 (2.95)	101.15 (2.95)	100.32 (2.97)
	M4	101.23 (3.03)	100.74 (2.99)	98.48 (2.97)	96.83 (2.99)
	M9	101.32 (3.05)	99.98 (3.00)	97.60 (2.99)	93.73 (2.99)†
P value	BL vs. M4	0.32	0.23	0.003	<0.0001
	BL vs, M9	0.39	0.05	0.0002	<0.0001
Body mass index‡	BL	34.52 (0.91)	34.54 (0.90)	34.73 (0.90)	34.39 (0.91)
	M4	34.12 (0.93)	34.21 (0.92)	33.83 (0.91)	33.13 (0.91)
	M9	34.16 (0.94)	33.84 (0.92)	33.56 (0.92)	32.11 (0.92)
P value	BL vs. M4	0.25	0.31	0.003	<0.0001
	BL vs, M9	0.32	0.03	0.0005	<0.0001
Waist Circumference (cm)‡	BL	106.26 (2.19)	108.29 (2.18)	105.91 (2.18)	106.04 (2.19)
	M4	104.69 (2.26)	107.10 (2.23)	102.99 (2.21)	102.12 (2.21)
	M9	102.77 (2.28)	105.87 (2.24)	102.32 (2.23)	99.27 (2.22)
P value	BL vs. M4	0.09	0.17	0.0005	<0.0001
	BL vs, M9	0.0004	0.008	<0.0001	<0.0001
% Body Fat	Baseline	36.37 (0.76)	36.69 (0.75)	36.57 (0.75)	36.46 (0.75)
	Month 4	34.40 (0.87)	33.19 (0.83)	33.17 (0.81)	32.93 (0.79)
	Month 9	33.12 (0.92)	32.37 (0.86)	32.38 (0.85)	31.42 (0.81)
P value	BL vs. M4	0.0002	<0.0001	<0.0001	<0.0001
	BL vs, M9	0.0001	<0.0001	<0.0001	<0.0001

BL, baseline; M4, month 4; M9, month 9; s.e., standard error.

*Estimates adjusted for age, gender, race, education, energy expenditure, and recruitment wave.

†Significantly different from Standard Care.

‡Significant group*time interaction (all P ≤ 0.01)

The initial waist circumference results were comparable to the weight loss results in that all 3 intervention groups had significant reductions in waist circumference at month 9 (Table 2). The major difference was that the Standard Care group also had a significant waist circumference reduction at month 9 (P = 0.0004). Two participants in this group, compared to baseline measurements, lost more than 12 cm during the 9 month study. After reviewing the feedback survey that the participants completed during the month 9 visit, we found that these two participants were strongly motivated to lose weight when they joined the study. Even though they were assigned to the Standard Care group, they tracked their daily diet and activity and weighed themselves daily. After removing these two participants from the analysis, the observed waist circumference reduction at month 9 for the Standard Care group was no longer significant. Therefore, we believe the large waist circumference reduction observed in these two participants is the main reason for the significant difference found in the Standard Care group at month 9. When compared to the Standard Care group, no significant waist circumference differences were found at month 9. No group differences in BMI were observed among intervention and Standard Care groups at month 9.

There were significant percent body fat reductions in all 4 study groups at month 9 (Table 2). However, no group differences in percent body fat were observed between the intervention and Standard Care groups.

Finally, we compared participants who received a SWA (GWL + SWA and SWA alone) with those who did not receive a SWA and found that, at month 9, those with an Armband showed significant weight and waist circumference reductions compared to those without an Armband.

## Discussion

The primary aim of this study was to evaluate how continuous self-monitoring using the SWA affected weight loss and waist circumference reduction both alone and in combination with GWL in sedentary overweight or obese adults. We demonstrated that a 9-month lifestyle intervention with the SWA produced significant weight loss and reduction in waist circumference. Thus, the SWA approaches, including real-time feedback and self-monitoring of energy balance, appeared to be beneficial tools in weight loss intervention. To our knowledge, only one other study [21] has evaluated weight loss in overweight and obese adults using a lifestyle intervention based on self-monitoring through the SWA.

The goal behind the design and development of the SWA was to create a wearable device that could quantitatively assess energy balance, sleep, and physical activity in free-living environments more efficiently and effectively than current alternatives. With regard to weight loss programs, SWA offers valuable assistance with the goal to simply self-monitor the physical activity and caloric intake during an intervention. In fact, the adherence to armband wear was excellent. Among the 76 participants assigned to an Armband group (SWA-alone and GWL+SWA) and who completed month 4 assessments, 75% wore the armband more than 75% of days. Those who wore the armband more than 75% of days experienced significant weight loss compared with those who wore it less than 75% of days [22]. Self-monitoring, a "cornerstone" of behavioral treatment, has been found to be correlated significantly with weekly weight loss [23]. However, due to the variability in adherence measurement methods, it is difficult to compare adherence across studies [23]. Burke and colleagues reported a median 55% adherence to standard self-monitoring with a paper diary [24,25]. In our study, adherence to armband wear was higher than 55%, suggesting that weight loss participants may better adhere to self-monitoring protocols that use technology, compared to standard protocols.

Our finding that self-monitoring of diet and physical activity with the SWA was related to successful weight loss supports previous studies that have identified the value of self-monitoring in weight loss [23,26]. A recent paper [23] presents a systematic review of the literature on three components of self-monitoring in behavioral weight loss studies: diet, exercise, and self-weighing. The use of technology, which included the internet, personal digital assistants, and electronic digital scales, was reported in five of the 22 identified studies. A significant association between self-monitoring and weight loss was consistently identified. One unique aspect of our study was that the self-monitoring was continuous and in "real-time". Recent systematic reviews of randomized controlled trials of weight loss [27,28] have concluded that weight loss interventions can be effectively delivered over the Internet. Successful online obesity treatment programs have targeted reduced energy intake, increased physical activity, and cognitive-behavioral strategies including personalized feedback, self-monitoring, and social support. However, limitations of previous studies include no intention-to-treat (ITT) analysis, no assessor blinding, follow-up measures based only on participants' self-report, moderate retention rates, and insufficient follow-up.

In a recent study the armband was used as a real-time self-monitoring device in conjunction with a GWL [21]. This small study (n = 57) reported improved weight loss over 3 months when the armband was worn in conjunction with a GWL beyond that which was accomplished with a GWL alone [21]. Polzien and colleagues' focus was to evaluate the efficacy of providing real-time feedback on energy balance as part of a weight reduction program, and to evaluate whether this approach enhances standard weight loss methods. Research suggests that individuals who use self-monitoring strategies (i.e., frequent weight checks, monitoring physical activity, monitoring caloric intake) experience improved weight loss [4,29]. Our study also showed a significant weight loss at month 9 for participants who wore a SWA compared with those who did not. These findings further confirm the importance of self-monitoring, which is indeed a consistent predictor of successful weight loss in technology-assisted weight management programs [29,30].

Due to the public health importance of overweight and obesity, weight loss interventions must be effective, available and accessible to the public. The Internet and technology devices provide unique opportunities for developing and implementing of lifestyle interventions that promote self-monitoring [31-33]. One study reported that overweight participants who had access to an Internet behavioral weight loss program for 6 months showed greater weight loss and reductions in waist circumference than participants given access to a weight loss education material only [34].

Our study had several strengths: a randomized design, primary and secondary outcomes assessed, including objective measures of adiposity, outcomes assessed by researchers blinded to group assignment, a participant population comparable to South Carolina's racial profile (67% white) and follow-up assessments at months 4 and 9. The significant weight loss results from SWA-alone group are likely to be applicable outside the research setting because participants in this group received minimal face-to-face intervention, prepared all their own meals, and established their own physical activity regimens.

There were also several limitations in the present study. First, there was a large attrition rate, particularly from the Standard Care group, where only 52% of the initial sample had complete data at month 9. Although the attrition rate is disappointing, it does not diminish our findings. Those lost to follow-up were similar to those who completed the study with the exception of a difference in education levels. Moreover, since we assumed no weight loss occurred in individuals lost to follow-up (initial weights carried forward), attrition biases our results toward finding no effect rather than overstating the effects of our interventions. Future studies are warranted to confirm or reject the findings reported here. Second, the participant sample was mostly female (82%) and highly educated (77%) and therefore may not be generalizable to the general populations, but is representative of individuals typically seeking weight loss treatment[35-37]. Third, this was a short intervention with significant weight loss, and therefore, it is unknown whether the weight loss would be maintained or continued over long term. Lastly, the GWL did not perform well compared with the published studies. One possibility is that participants were disappointed with group assignment. Another possible cause is the different characteristics of participants recruited during different waves. The first wave of recruitment was mainly from the University employees and students. We later found out that students were the most unreliable group in this study, and their adherence was especially poor for homework assignments and other assignments.

## Conclusions

Finding new and innovative ways to use technology to reach individuals who are seeking weight loss via lifestyle interventions is vital. Long-term studies should be carried out to see if the SWA device can produce long-lasting effects or if combining the SWA and GWL (monitoring, feedback, and support) enhances weight loss compared to either intervention modality alone. Therefore, future studies should continue to refine applications technology and its ability to further enhance healthy lifestyle change for weight loss. This may provide an opportunity to objectively and accurately assess free-living energy balance, and therefore enhance the understanding of the contribution of energy expenditure/intake to weight loss.

## Competing interests

This study was funded by an unrestricted research grant from BodyMedia, Inc to Steven N. Blair, Principal Investigator. Dr. Blair and the research team at the University of South Carolina planned and executed the study, analyzed the data, and wrote the manuscript. None of the members of the research team own any shares in BodyMedia, Inc; and none of them hold patents, nor are they applying for any patents related to this research.

Dr. Blair receives book royalties (<$5,000/year) from Human Kinetics; honoraria for service on the Scientific/Medical Advisory Boards for Alere, Technogym, Santech, and Jenny Craig; and honoraria for lectures and consultations from scientific, educational, and lay groups. During the past 5-year period he has received research grants from the National Institutes of Health, Department of Defense, and Coca Cola. None of the other authors has any competing interests to declare.

## Authors' contributions

SNB was Principal Investigator for this study. SLS was the lead writer on the manuscript. AM and SW contributed to developing the intervention. SLS, VWB, XS contributed to the development of the data collection procedures. XS and JWH contributed to the statistical analysis of the data. GAH, SW, RAM, SNB, and XS contributed to the interpretation of findings. All authors critiqued and edited drafts of the manuscript and approved the submitted version.
